# Parasites as Biological Tags for Stock Discrimination of Beaked Redfish (*Sebastes mentella*): Parasite Infra-Communities vs. Limited Resolution of Cytochrome Markers

**DOI:** 10.1371/journal.pone.0153964

**Published:** 2016-04-22

**Authors:** Regina Klapper, Judith Kochmann, Robert B. O’Hara, Horst Karl, Thomas Kuhn

**Affiliations:** 1 Department of Safety and Quality of Milk and Fish Products, Federal Research Institute of Nutrition and Food, Max Rubner-Institute, Hamburg, Germany; 2 Goethe-University, Institute for Ecology, Evolution and Diversity, Frankfurt am Main, Germany; 3 Senckenberg Gesellschaft für Naturforschung, Senckenberg Biodiversity and Climate Research Centre, Frankfurt am Main, Germany; Universidade de Aveiro, PORTUGAL

## Abstract

The use of parasites as biological tags for discrimination of fish stocks has become a commonly used approach in fisheries management. Metazoan parasite community analysis and anisakid nematode population genetics based on a mitochondrial cytochrome marker were applied in order to assess the usefulness of the two parasitological methods for stock discrimination of beaked redfish *Sebastes mentella* of three fishing grounds in the North East Atlantic. Multivariate, model-based approaches demonstrated that the metazoan parasite fauna of beaked redfish from East Greenland differed from Tampen, northern North Sea, and Bear Island, Barents Sea. A joint model (latent variable model) was used to estimate the effects of covariates on parasite species and identified four parasite species as main source of differences among fishing grounds; namely *Chondracanthus nodosus*, *Anisakis simplex* s.s., *Hysterothylacium aduncum*, and *Bothriocephalus scorpii*. Due to its high abundance and differences between fishing grounds, *Anisakis simplex* s.s. was considered as a major biological tag for host stock differentiation. Whilst the sole examination of *Anisakis simplex* s.s. on a population genetic level is only of limited use, anisakid nematodes (in particular, *A*. *simplex* s.s.) can serve as biological tags on a parasite community level. This study confirmed the use of multivariate analyses as a tool to evaluate parasite infra-communities and to identify parasite species that might serve as biological tags. The present study suggests that *S*. *mentella* in the northern North Sea and Barents Sea is not sub-structured.

## Introduction

Stock identification is a key component for the management of economically important fish species as it improves the understanding of the vulnerability of unequally exploited subpopulations within a species range and thus, helps the implementation of sustainable fisheries [1–3]. Non-consideration of stock structure may lead to decreases in stock abundances, genetic diversity, and changes in biological attributes [4]. Hence, correct identification of stocks is a prerequisite in order to prevent overfishing and depletion of less productive stocks [1,5,6].

Beaked redfish *Sebastes mentella* (Travin, 1951) is a major commercially exploited fish species in the North Atlantic. Redfish (*Sebaste*s spp., mainly of *S*. *norvegicus* and *S*. *mentella*) is primarily fished in the northeastern Irminger Sea with annual landings ranging between 30000 and 70000 t since 2005, while along the coast of Norway up to the Barents Sea annual landings are about 10000 t, mainly as bycatch [7]. Special characteristics in reproductive biology, early life history, and longevity result in a complex inter- and intra-species population structure of *Sebastes* spp. making their stocks vulnerable and slow in recovery from fishing pressure [7–9]. *Sebastes mentella* has a long life span, grows slowly, and matures lately. It is ovoviviparous [7], and has a planktivorous diet, with Copepoda, Euphausiacea, Mollusca, Decapoda, and Myctophidae as main sources of food [10–12]. *Sebastes mentella* is distributed across the North Atlantic in deep waters from 300–750 m, but it can also be found at depths of 1,000 m [13–15]. According to the International Council for the Exploration of the Sea (ICES) three stocks are recognized in the Irminger Sea and adjacent waters [16], which include a deep-pelagic stock below 500 m from Labrador to Faroe Islands, a shallow-pelagic stock above 500 m from Grand Bank to Faroe Islands, and an Iceland slope stock [8]. However, the population structure in the Barents Sea and Norwegian Sea is still unresolved, but it has been suggested that they form one distinct stock [7,8].

Stock identification is mainly based on data from morphological characteristics, genetic techniques, artificial and biological tags [7,8]. The latter also includes an approach to determine fish stock structuring by the use of parasites as biological indicators. As such, parasites can give indications on host biology, intraspecific population dynamics, and individual origin [17–19]. Parasites as biological tags for redfish have a long tradition in their use for stock discrimination, e.g. the crustaceans *Sphyrion lumpi* and *Chondracanthus nodosus*, and the nematode *Anisakis simplex* [18,20–23]. The genetic variability and spatial structure of parasite populations may also reflect the population structure of their host [24,25]. As parasite populations may diverge faster than host populations [2], parasites can more successfully assign the hosts to their population of origin [26]. Therefore, an increasingly common approach for biological tags is the use of parasite genetic structuring [27,28]. For anisakid nematodes knowledge on the population structure is still scarce [29].

The aim of the present study was to evaluate the usefulness of parasites as biological tags for host stock differentiation of the commercially important beaked redfish of three fishing grounds from the North East Atlantic. As parasite assemblage data have been successfully applied in assessments of other fish populations [30–32], and parasite population genetics analyses showed good potential as they were more sub-structured than their hosts [24,28,33], a combination of data on metazoan parasite community and parasite population genetic structure was chosen in this study. Multivariate analyses, in particular a latent variable model (LVM) as an extension of a generalized linear model was fitted to estimate the effects of covariates on the abundance of parasite species and a model-based ordination to visualize fishing grounds and parasite species patterns was used. To our knowledge, this is the first study that uses this recent approach [34] for parasite infra-community analysis. Haplotype structure of the widespread nematode *A*. *simplex* s.s. was assessed using cytochrome c oxidase 2 (*cox2*) to test whether a genetic population differentiation would be possible.

## Materials and Methods

### Ethics statement

Approval of our present study by a review board institution or ethics committee was not necessary because all fish were caught during a regular research cruise on board of the FRV Walther Herwig III. The study was conducted according to the governmental permission of the corresponding territorial waters: Maritime Policy Unit of the Foreign Commonwealth Office (Tampen), Norwegian Directorate of Fisheries (Bear Island), and Danish Ministry of Foreign Affairs (East Greenland). No living animals were used. All fish were expertly killed according to the German Animal Protection Law (§4) and the ordinance of slaughter and killing of animals (*Tierschlachtverordnung* §13). The species is neither endangered nor protected.

### Sample collection

**Fig 1:**
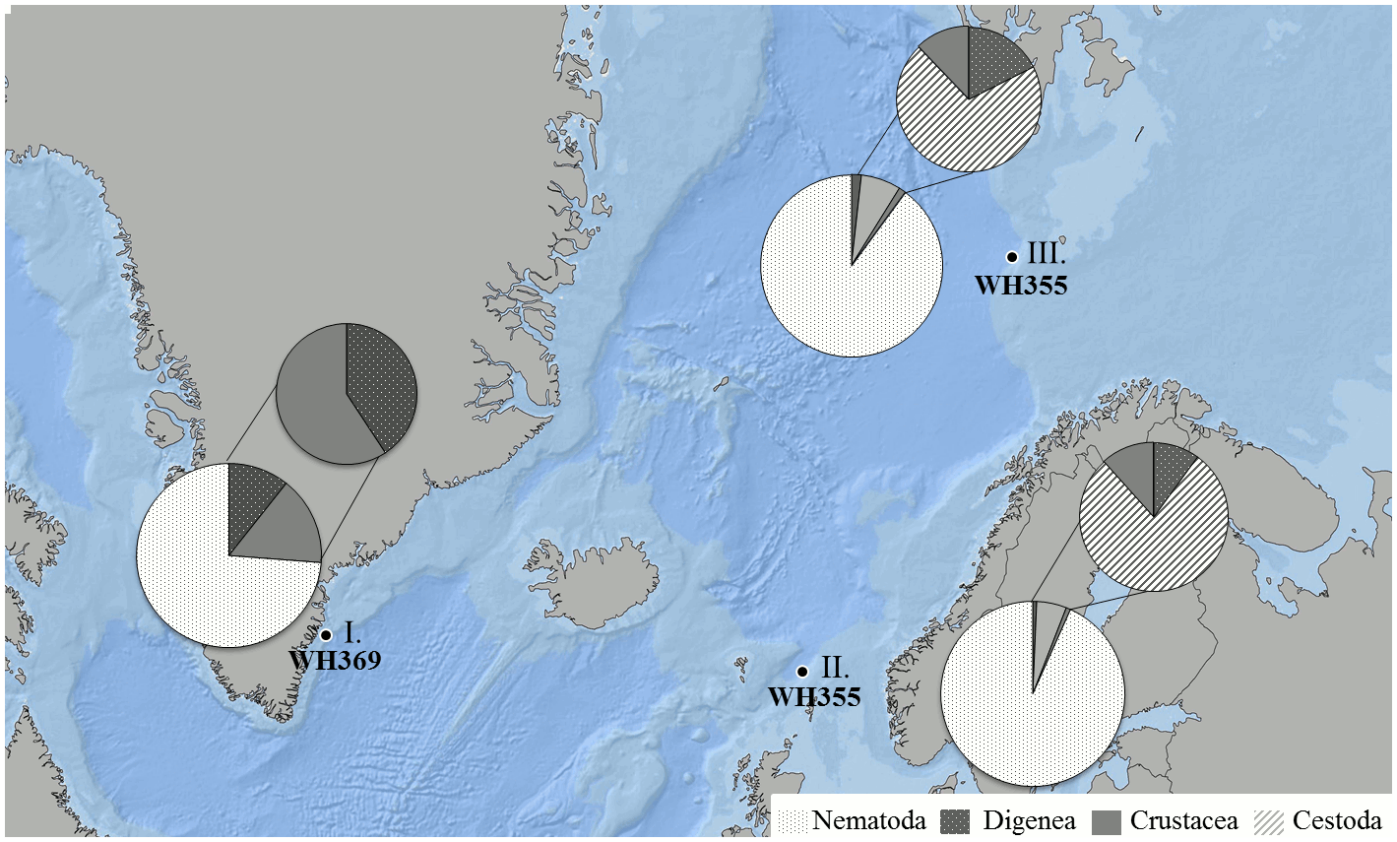
Map of fishing grounds with relative distributions of parasite groups. Large pie charts: all parasite groups, small pie charts: proportion of parasite groups excluding Nematoda. I: East Greenland, II: Tampen, northern North Sea, III: Bear Island, Barents Sea. The map was modified from Klapper et al. [35] and is for illustrative purposes only. Map Source: GIS.

Fourty *Sebastes mentella* from each of the three sampling stations (Tampen: northern North Sea; Bear Island: Barents Sea, East Greenland) were collected on board of the German FRV Walther Herwig III during the research cruises WH355 (Tampen: Northern North Sea; Bear Island: Barents Sea) in June 2012 and WH369 (East Greenland: Irminger Sea) October 2013 (Fig 1). Fish were caught with bottom trawls from Tampen (62°04.27’N 000°00.61’W), from Bear Island (74°06.72’N, 016°25.21’E), and from Greenland (63°26.98’N, 039°07.55’W), and deep frozen at -30°C until examination.

### Parasitological examination

Host biometric parameters were measured for each individual. Total length (TL) was measured to the nearest centimetre and total weight without stomach content (TW) to the nearest gram. The gills, nostrils, skin, fins, eyes, and mouth cavity were examined macroscopically for ectoparasites. Fish were opened, internal organs were removed, separated and transferred into petri dishes filled with 0.9% saline solution. Liver, gonads, filled and empty stomachs were weighed to the nearest gram. The liver, gonads, stomach, gut, gall bladder, pyloric caeca were dissected and microscopically examined for endoparasites with a magnification of 6.7–45 x (Olympus SZ 61, Germany). Fish fillets and belly flaps were examined for anisakid nematodes via UV-press method as described by Karl and Leinemann [36]. Host tissue was removed from isolated parasites. Encysted nematode larvae were released from their cysts. Digenea, Cestoda, Crustacea, and some nematodes were fixed in 4% borax buffered formalin, and preserved in 70% ethanol. Nematode larvae were preserved with 4% glycerol for morphological identification. For molecular identification, the remaining anisakids were stored in absolute ethanol. Terminology for parasitological and ecological examination followed Bush et al. [37]. Original descriptions were used for parasite identification [38–42]. Nematodes were identified morphologically to genus level and subsamples of *Anisakis* spp., *Pseudoterranova* spp., and *Hysterothylacium* spp. were identified using standard molecular methods (see below).

### Molecular nematode identification

For molecular identification of the nematode species, random subsamples from all fishing grounds of *Hysterothylacium* spp. (n = 9), *Pseudoterranova* spp. (n = 1), and *Anisakis* spp. larvae (n = 4) were taken, and the rDNA region comprising the ITS-1, 5.8S, ITS-2 and flanking sequences (ITS+) was used. DNA was isolated and purified from nematode larvae using the peqGOLD Tissue DNA Mini Kit (Peqlab Biotechnology GmbH, Erlangen, Germany) according to the instructions of the manufacturer. The ITS PCR-reaction (25 μl) for the amplification of primers TKI (5'-GGC-AAA-AGT-CGT-AAC-AAG-GT-3') and NC2 (5'-TTA-GTT-TCT-TTT-CCT-CCG-CT-3') [43,44] included 12.5 μl Master-Mix (Peqlab Biotechnology GmbH, Erlangen, Germany) containing dNTP, MgCl_2_, Buffer and Taq-Polymerase, 1.5 μl of each primer (10 pmol μl^-1^), 14 μl ddH_2_O, and 2.5 μl DNA. The PCR cycling parameters included the following conditions: initial denaturation at 94°C for 120 sec, 40 cycles of 94°C for 20 sec (denaturation), 51°C for 20 sec (annealing), 72°C for 50 sec (extension), followed by a final extension at 72°C for 5 min in a thermocycler (Eppendorf, Germany).

To specifically test for population differentiation *Anisakis* spp. larvae (n = 80) were identified and haplotypes were later analysed by the use of mitochondrial DNA (mtDNA) of a fragment of the *cox2* gene region (with primers 210 and 211 as described in Nadler and Hudspeth [43]). The mtDNA PCR reactions (50 μl) contained 25 μl Master Mix which included dNTPs, MgCl_2_, Buffer and Taq-Polymerase, 3μl of each primer (10 pmol μl^-1^), 14 μl ddH_2_0, and 5 μl DNA. *cox2* PCR reactions were performed with an initial denaturation at 94°C for 90 sec, 40 cycles of 94°C for 30 sec (denaturation), 46°C for 60 sec (annealing), 72°C for 90 sec (extension), followed by a final extension at 72°C for 10 min in a thermocycler (Eppendorf, Germany).

Negative controls without DNA were added to each PCR run (rDNA, mtDNA) and PCR products were controlled on 1% agarose gels. To estimate the size of the PCR products a 100 bp ladder marker (peqGOLD, Erlangen, Germany) was used. Successfully amplified products were purified with the peqGOLD Cycle-Pure Kit (Peqlab Biotechnology GmbH, Erlangen, Germany) following the instructions of the manufacturer. The purified products were sequenced by Seqlab (Goettingen GmbH, Germany) using primer TKI, 210 and 211, respectively. Sequences were analysed using BioEdit 7.2.5 [45], followed by an alignment using Clustal X2 with the default parameters. Sequence data were compared with previously published Genbank data using the BLASTn algorithm to identify the parasite species [46].

### Data analyses

For univariate analysis, a generalized linear model (GLM) analysis (with negative binomial link) was done. The total parasite abundance of fish was taken as the response variable and total length of fish (TL), Condition Index of fish (Fulton’s K = 100(TW/TL³)), gender (female/male), and fishing ground were taken as potential predictors. The model included fishing grounds (Bear Island, Greenland, Tampen) and gender as categorical variables, and total length and the condition factor Fulton’s K as continuous variables. The model included only the main factor effects and no interaction terms. Covariates of host biometric values, abundances, and intensities are given with mean and standard deviation (±SD). Statistical analyses were conducted in R (version 3.0.2). Parasite fauna diversity was calculated by using the Shannon-Wiener (*H’*) and Pielous’s evenness (*E*) according to Magurran [47]. Furthermore, taxonomic distinctness was calculated. It measures the average degree to which species are related to each other [48,49] and is defined as the average taxonomic path length between any two randomly chosen species based on Linnean classification to reach a common taxon [48]. A greater value of taxonomic distinctness indicates a large average distinctness between species within a parasite assemblage [50]. The taxonomic levels used in this study were species, genus, family, order, subclass, class and phylum with taxonomies used from World Register of Marine Species (WoRMS, http://www.marinespecies.org). Taxonomic distinctness (Δ^+^) and the variance in taxonomic distinctness (Λ^+^) were calculated in PRIMER v.6.

A multivariate analysis was used to explore differences in metazoan parasite fauna among fishing grounds. A model-based ordination was carried out using the boral package v0.9.1 [34]. This fits a multivariate generalized linear model to the data, so we can model the effects of specific covariates (in this study location, total length and Fulton's K: location is taken as the difference between each location and Bear Island) on each species. Bayes factors were calculated and the strength of evidence of an effect was interpreted according to Kass and Raftery [51]. The residual co-variation between species was modelled using a latent model: based on the assumption that the covariation can be summarised in two dimensions, we fit a model with two latent variables. These were plotted in a biplot, which shows correlations between taxa that arise for reasons not attributable to measured predictors [52]. Stochastic search variable selection (SSVS) was used to test whether each variable had an effect: this shrinks small coefficients to be close to zero. Total length and Fulton's K were scaled to have variance 1 and the “slab” for the SSVS was given a prior variance of 10.

By using the parasite abundance data of this study, the probabilities for the significant parasite species in the parametric ordination were calculated for their use as biomarker. The presence of individual parasite species was used to classify the fish into population. If the proportion of fish in population *s* with a parasite is *p*_*s*_, and the number of fish in population *S* is *N*_*S*_ then the probability that a fish with a parasite comes from population *s* is *Pr*(*S* = *s|X*) which is (by simply applying Bayes' rule)

$$Pr\left(S=s|X\right)=\frac{Pr\left(X|S=s\right)Pr\left(S=s\right)}{\sum_{t}\left(X|S=t\right)Pr\left(S=t\right)}$$

where *Pr*(*X*/*S* = *s*) is simply the proportion of infected fish in population *s* and *Pr*(*S* = *s*) is the probability that a random fish comes from population *s*, i.e. *Pr*(*S* = *s*) = *N*_*s*_/Σ_*t*_ *N*_*t*_. If we assume equal probabilities (for simplicity), *Pr*(*S* = *s*) cancels out, so we can calculate this simply from the proportions of infected fish in each population. Note that two fish had to be excluded from the analyses as weight values were missing. Host biometric and parasite raw data are available from FigShare; the DOI is 10.6084/m9.figshare.2729782, R Markdown documents online on Rpubs (http://rpubs.com/oharar/170545).

Analyses of *cox2* sequences were performed with DNAsp 5.0 [53] to calculate the following statistics: number of unique haplotypes, haplotype diversity (*h*), nucleotide diversity (π). Tajima’s D and Fu’s F were conducted to test for selective neutrality and demographic processes [54,55]. Pairwise and overall distances among haplotype sequences were conducted using MEGA 5 [56]. Analysis of molecular variances (AMOVA) was performed with 10,000 permutations to test the within and between variation of fishing grounds. Pairwise genetic differentiation among fishing grounds were estimated using the fixation index (*F*_ST_), with 10,000 permutations. AMOVA, *F*_ST_, Fu’s F, and Tajima’s D were calculated using Arlequin 3.5.1.2 [57]. A median-joining haplotype network was created for *Anisakis simplex* s.s. to investigate genetic relationships among haplotypes from different fishing grounds using NETWORK 4.6.1.0 [58].

## Results

### Host biometric data

**Fig 2:**
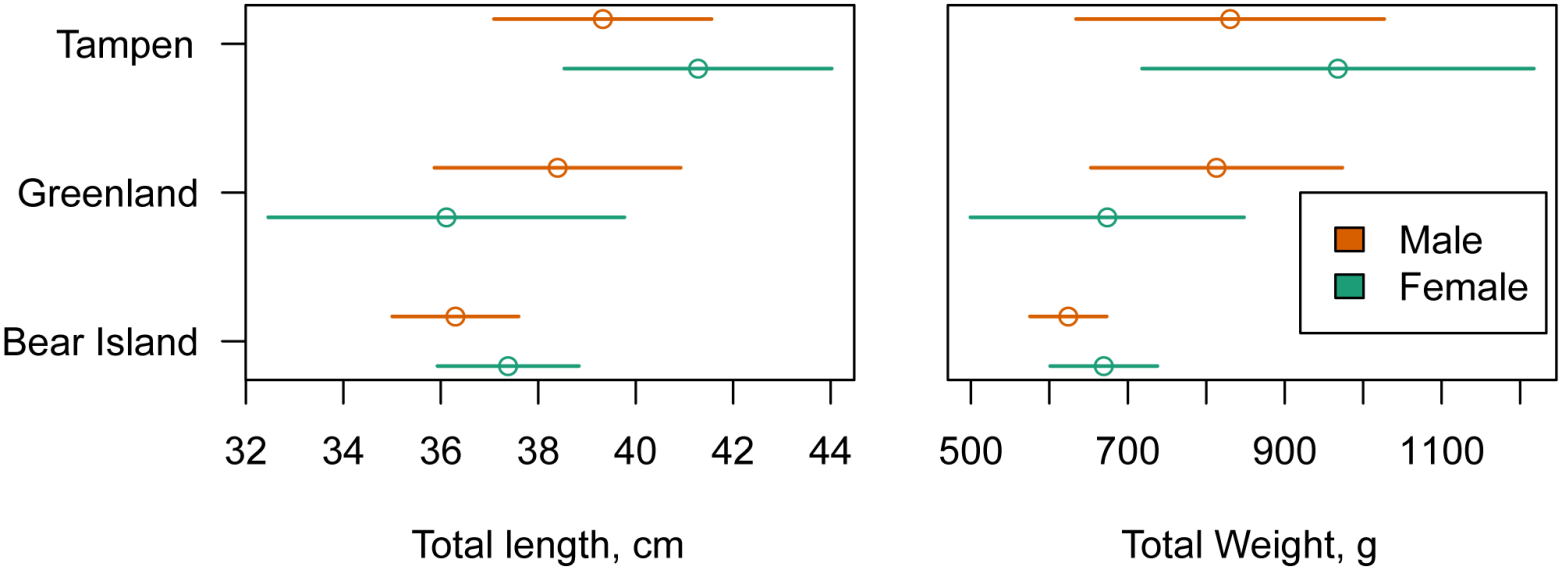
Host biometric data from three fishing grounds separated by gender. Means±standard deviation of the different covariates. Total weight given without stomach content.

Mean total length (TL) was 38.0 cm±3.0 (SD) (range 25.6–45.9), and mean total weight without stomach content (TW) 754.0 g±190.7 (181.0–1348.0.) from all samples examined. Standard length of fish was 32.3 cm±2.4 (range 21.3–34.7). The condition factor Fulton’s K, calculated without stomach content weight, was 1.34±0.12 (range 1.08–1.43). Fish from Tampen tended to be larger and heavier. Fish from Bear Island were smaller, but the overlap was high (Fig 2).

### Molecular nematode identification

14 anisakid nematode larvae were identified using ITS markers and 80 *Anisakis* spp. larvae using *cox2* markers. Sequences were aligned to reference sequences in GenBank. Determination of the sibling species was conducted if the alignment of the sequence data revealed a 98–100% identification (e-value: 0.00) with sequences in GenBank. The alignment revealed three nematode species: *Anisakis simplex* sensu stricto (GenBank- Acc.: *cox2*, n = 80: KT852457—KT852536, ITS, n = 4: KT852537- KT852540), *Hysterothylacium aduncum* (ITS, n = 9: KT852541- KT852549), and *Pseudoterranova decipiens* sensu stricto (ITS, n = 1: KT852550).

### Parasite assemblage analysis

**Fig 3:**
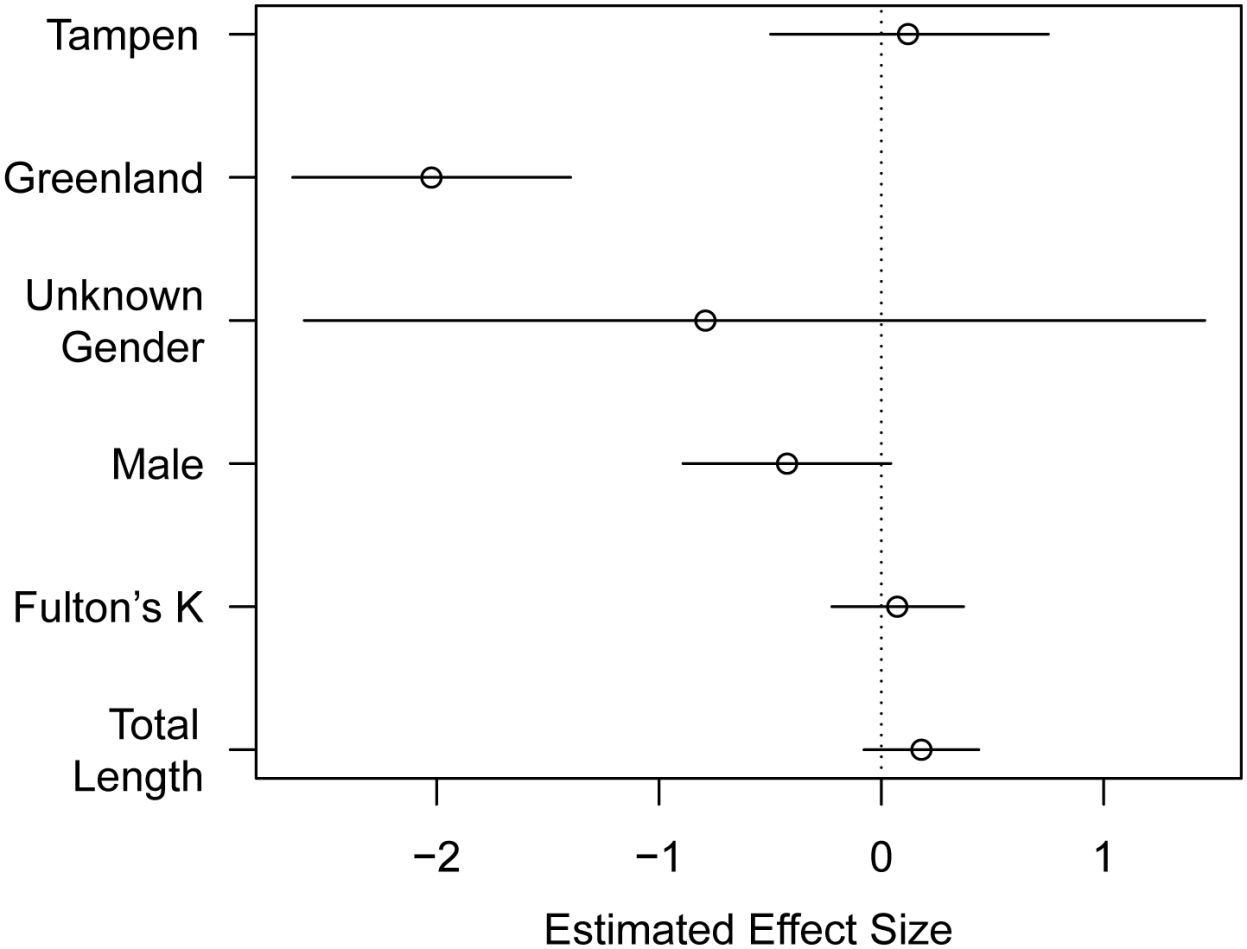
Estimated Coefficients for effects of covariates on total abundance of parasites. Fit of a generalized linear model (GLM) contrasted to Bear Island and female, assuming a negative binomial response. Note that total length and Fulton's K are standardised, so the coefficient represents the change in log abundance when the covariate (e.g. total length) changes by one standard deviation.

In total, 1735 parasites of 14 different species were recovered: Digenea (7 species), Nematoda (3 species), Cestoda (2 species), and Crustacea (2 species) (Table 1). Greenland proved to have a highly significant effect on average individual number of parasites (GLM), with no effect of gender, Fulton’s K, or total length (Fig 3).

**Table 1:** Infection parameters of beaked redfish for pooled and separated fish samples per fishing ground. N = Number of parasites. mA = mean abundance, P = prevalence, mI = mean intensity, SD = standard deviation.

	Total					Tampen			Bear Island			Greenland		
Parasite	N Parasite	mA ± SD	Range	P [%]	mI ± SD	mA ± SD	P [%]	mI ± SD	mA ± SD	P [%]	mI ± SD	mA ± SD	P [%]	mI ± SD
**Digenea**	**30**	**0.3 ± 0.6**	**0–4**	20	**1.2 ± 0.7**	**0.15 ± 0.36**	**15**	**1**	**0.33 ± 0.73**	**25**	**1.30 ± 0.95**	**0.28 ± 0.60**	**20**	**1.38 ± 0.52**
Digenea indet.	5	0 ± 0.2	0–1	4	1	0.08 ± 0.27	7.5	1	0.05 ± 0.22	5	1	0	0	-
*Gonocerca phycidis*	1	0 ± 0.1	0–1	1	1	0	0	-	0.03 ± 0.16	2.5	1	0	0	-
*Derogenes varicus*	6	0.1 ± 0.2	0–1	5	1	0	0	-	0.10 ± 0.30	10	1	0.05 ± 0.22	5	1
*Anomalotrema koiae*	10	0.1 ± 0.4	0–4	6	1.4 ± 1.1	0.08 ± 0.27	7.5	1	0.10 ± 0.63	2.5	4	0.08 ± 0.27	7.5	1
*Lecithophyllum botryophorum*	1	0 ± 0.1	0–1	1	1	0	0	-	0.03 ± 0.16	2.5	1	0	0	-
*Hemiurus sp*.	1	0 ± 0.1	0–1	1	1	0	0	-	0	0	-	0.03 ± 0.16	2.5	1
*Podocotyle reflexa*	4	0 ± 0.2	0–2	3	1 ± 0.7	0	0	-	0	0	-	0.10 ± 0.38	7.5	-
*Progonus muelleri*	1	0 ± 0.1	0–1	1	1	0	0	-	0	0	-	0.03 ± 0.16	2.5	1
**Nematoda**	**1590**	**13.3 ± 22.5**	**0–166**	87	**15.1 ± 23.5**	**20.95 ± 23.12**	**95**	**22.05 ± 23.21**	**16.50 ± 28.24**	**100**	**16.50 ± 28.24**	**1.90 ± 2.78**	**65**	**1.33 ± 0.58**
*Anisakis simplex* s.s.	1464	12.2 ± 22.5	0–166	78	15.6 ± 24.5	20.00 ± 23.59	85	23.53 ± 23.92	15.23 ± 28.19	100	15.23 ± 28.19	1.38 ± 2.78	47.5	2.92 ± 2.98
*Hysterothylacium aduncum*	54	0.5± 1.1	0–6	23	1.9± 1.4	0.35 ± 1.03	20	1.75 ± 1.75	0.85 ± 1.42	35	2.43 ± 1.40	0.15 ± 0.36	15	2.89 ± 3.48
*Pseudoterranova decipiens* s.s.	9	0.1 ± 0.3	0–2	7	1.1 ± 0.4	0.08 ± 0.27	7.5	1	0.13 ± 0.40	10	1.25 ± 0.50	0.30 ± 0.16	2.5	1
**Cestoda**	**99**	**0.8 ± 3.4**	**0–20**	18	**1.9 ± 3.3**	**1.18 ± 4.39**	**22.5**	**5.22 ± 8.38**	**1.30 ± 3.96**	**30**	**4.33 ± 6.41**	**0**	**0**	**-**
*Scolex pleuronectis*	82	0.7 ± 3.4	0–20	4	4.4 ± 1.4	1.00 ± 4.41	5	20	1.05 ± 3.92	7.5	14.00 ± 5.29	0	0	-
*Bothriocephalus scorpii*	20	0.2 ± 0.4	0–1	17	1	0.23 ± 0.42	22.5	1	0.28 ± 0.45	27.5	1	0	0	-
**Crustacea**	**32**	**0.3 ± 0.7**	**0–4**	17	**1.6 ± 1.0**	**0.18 ± 0.45**	**15**	**1.17 ± 0.41**	**0.23 ± 0.77**	**10**	**2.25 ± 1.26**	**0.40 ± 0.87**	**25**	**1.60 ± 1.07**
*Sphyrion lumpi*	17	0.1 ± 0.5	0–4	9	1.5 ± 0.9	0.18 ± ± 0.45	15	1.17 ± 0.41	0.23 ± 0.77	10	2.25 ± 1.26	0.03 ± 0.16	2.5	1
*Chondracanthus nodosus*	15	0.1 ± 0.5	0–1	8	1.7 ± 1.1	0	0	-	0	0	-	0.38 ± 0.87	22.5	1.67 ± 1.12

Nematodes were the predominant parasites with the highest abundances (Table 1, Fig 1). *Anisakis simplex* s.s. was by far the most abundant species. *A*. *simplex* s.s. were isolated from both intestines and musculature of *S*. *mentella* from the three fishing grounds, while *H*. *aduncum* and *P*. *decipiens* s.s. were recovered only from the intestines from all fishing grounds. The most diverse taxon with seven species was Digenea, which were located in the gastrointestinal tract of the redfish. Shannon-Wiener index (*H’*) and Pielous’s evenness (*E*) were highest in Greenland (*H’* = 1.320, *E* = 0.487), followed by Bear Island (*H’* = 0.698, *E* = 0.258), and lowest in Tampen (*H’* = 0.437, *E* = 0.161). The mean value of the average taxonomic distinctness was highest in Bear Island (Δ^+^ = 53.6, Λ^+^ = 109.8), followed by Tampen (Δ^+^ = 36.0, Λ^+^ = 113.0), and Greenland (Δ^+^ = 33.7, Λ^+^ = 82.8).

**Fig 4:**
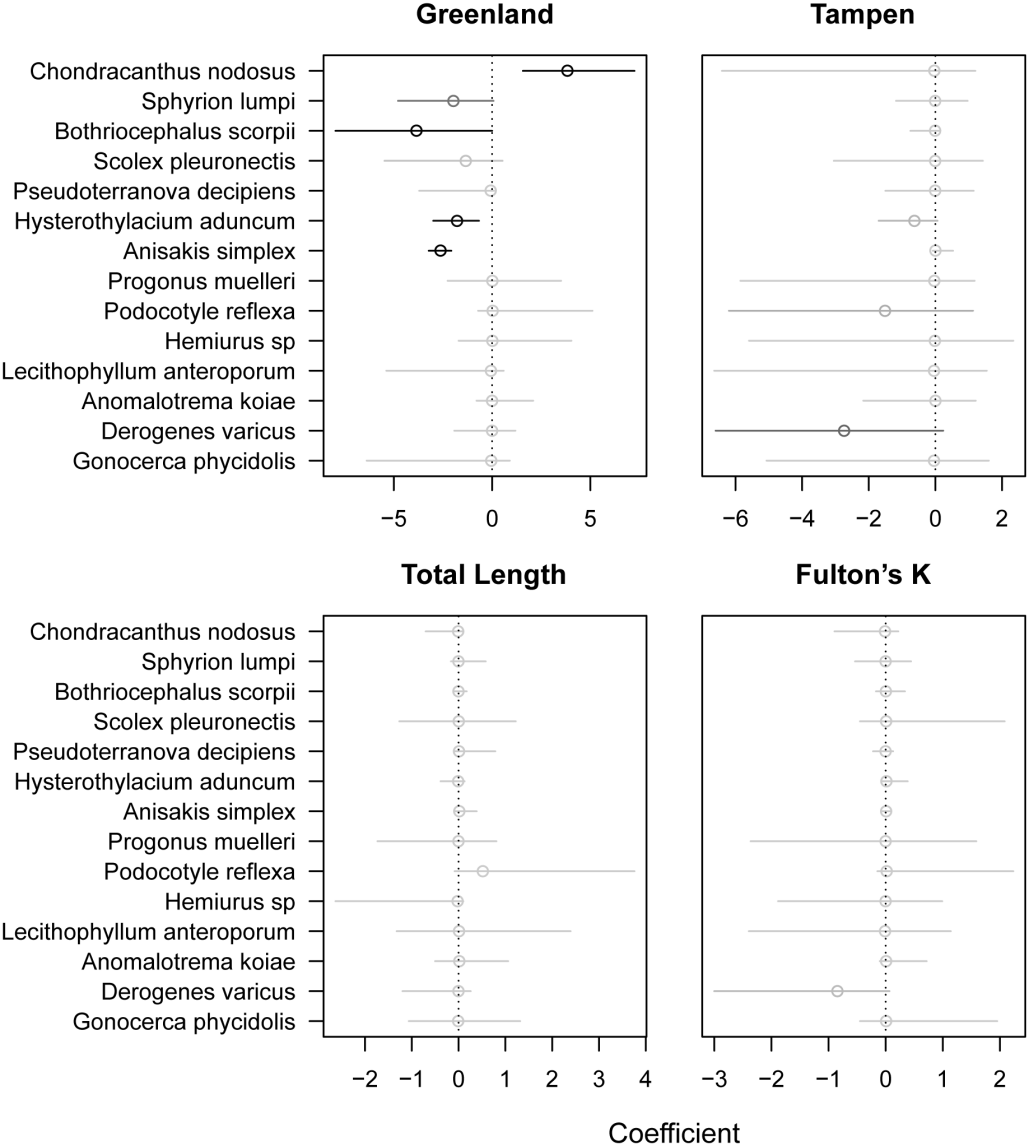
Posterior estimates of effects of covariates on the abundance of parasite species. Points: posterior mean, Lines: 95% Highest Posterior Density Interval. Fishing grounds Greenland and Tampen are contrasted to Bear Island. The darker the estimate, the higher the evidence of an effect.

The parametric ordination showed that only the difference between Greenland and Bear Island affected the community composition (Fig 4). Bayes factors (*K)* support strong evidence of effects for four parasite species: Bayes factors for *Chondracanthus nodosus (K* = 98) and *Hysterothylacium aduncum (K* = 65) were strong; *Anisakis simplex (K* = ∞) and *Bothriocephalus scorpii (K* = 197) provided strong to decisive evidence of an effect. Bayes factors were less than *K* = 3 for all other species. There was also no effect for total length and Fulton’s K.

**Fig 5:**
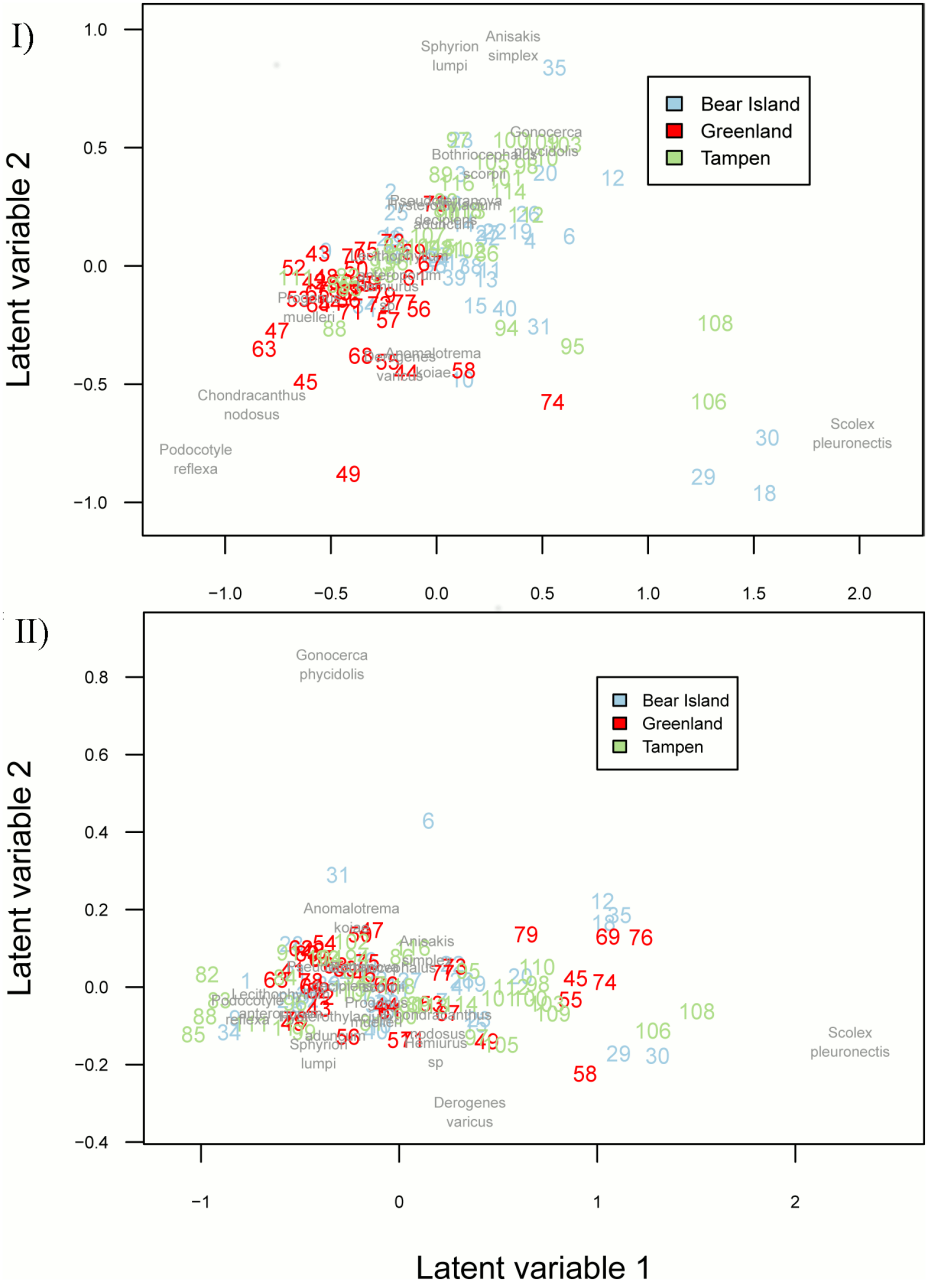
Model-based unconstrained and residual ordination biplot for the *S*. *mentella* parasite community among fishing grounds. Latent variable model (with two latent variables). I) Unconstrained ordination with no predictors: Fish of Greenland clusters apart from fish of the other two locations II) Residual ordination after controlling for the effect of fishing grounds: No visible pattern. Each number represents one fish specimen with the colours depicting its origin. Parasite species and fish specimen in the same direction are highly correlated.

The effect of fishing grounds is visualized in the ordination biplot, with fish from Greenland being different from the other fishing grounds (Fig 5I). In a partial ordination, in which the effect of fishing grounds was corrected, there was no pattern anymore, i.e. no shared response to environmental variables (Fig 5II). There is a pattern of *Scolex pleuronectis* sitting out from the other species.

### Biomarkers

Significant parasite species of the parametric ordination were used to estimate their use as biomarker (Table 2). The presence of *C*. *nodosus* had a 100% probability that a fish must be from Greenland. For *B*. *scorpii* the probability was 0% for Greenland. The probabilities that an observed nematode of an analysed fish stems from Greenland was lower than for the other two fishing grounds.

**Table 2:** Probabilities to assign the correct origin of a fish according to the presence of an identified parasite species.

	Bear Island	Greenland	Tampen
*Anisakis simplex*	0.42	0.20	0.38
*Hysterothylacium aduncum*	0.54	0.17	0.29
*Bothriocephalus scorpii*	0.52	0.00	0.48
*Chondracanthus nodosus*	0.00	1.00	0.00

### Genetic structure of *Anisakis simplex*

**Fig 6:**
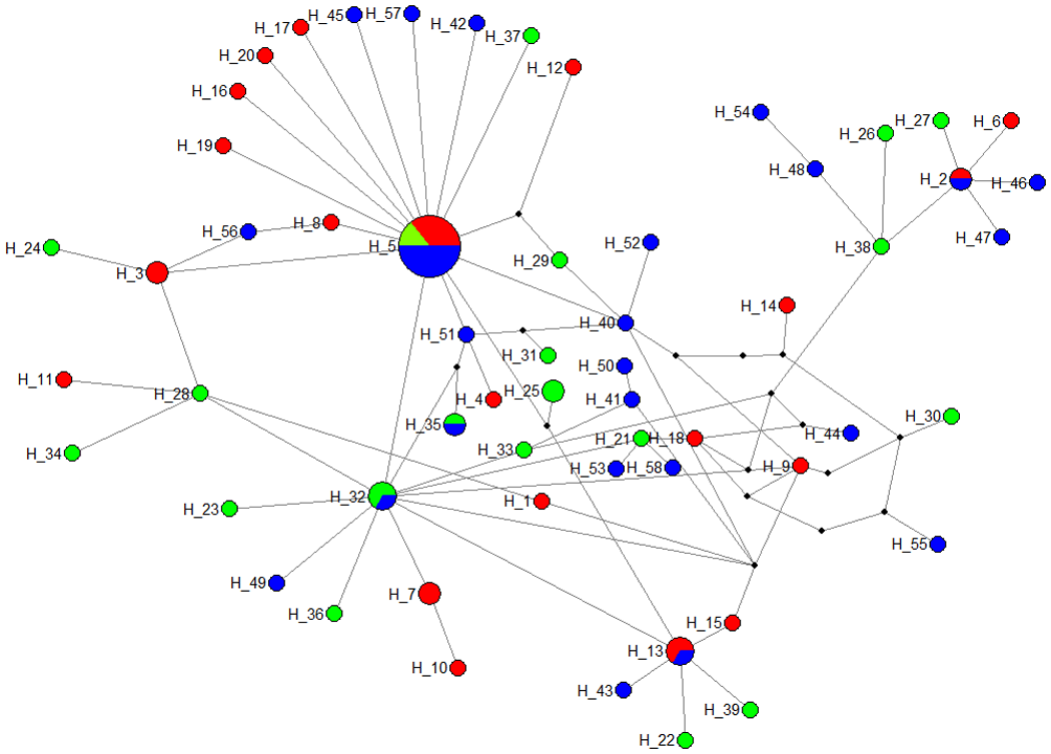
Median joining network of *Anisakis simplex* s.s haplotypes from *cox2* mitochondrial DNA sequences (n = 80), inferred from statistical parsimony. The size of pie charts corresponds to the frequency of haplotypes. Black nodes represent hypothetical haplotypes that were required for the establishment of the sampled haplotypes. Blue = Greenland, red = Tampen, green = Bear Island, H = haplotype with the according number.

Population structure of *Anisakis simplex* s.s. was assessed by using *cox2* mtDNA sequences with a length of 582 bp recovered from 80 nematode larvae. Number of variable sites was 52 and 58 haplotypes were detected from the *cox2* fragments. Of the 58 haplotypes obtained, 50 were unique, i.e. occurred only once in the dataset (86.2%). There was a lack of geographic structuring in the parsimony network analysis (Fig 6). The parsimony haplotype network was star-shaped with the most common haplotype (H5) in the middle. The most abundant haplotype H5 was present in 14 individuals from all three fishing grounds. Two haplotypes were shared between Tampen and Greenland (H2, H13), and Bear Island and Greenland (H32, H35). Genetic diversity indices for all fishing grounds and for pooled samples are shown on Table 3. Haplotype diversities ranged from 0.952 to 0.988 and the overall haplotype diversity (*h*) was 0.967. Nucleotide diversity ranged from 0.006 to 0.007. Tajima's D was negative for all fishing grounds, with a significant overall Tajima’s D = -1.636 (p = 0.03). Also Fu’s F was negative and the null hypothesis, i.e. the population evolves according to the infinite-site model and all mutations are selectively neutral, was rejected (Fu’s F = -16.975, p<0.01). Mean pairwise sequence divergence was 0.007 (Tamura-Nei distance), and mean pairwise divergence among groups was 0.008 for Bear Island and Greenland, 0.007 for Bear Island and Tampen, and 0.007 for Tampen and Greenland. AMOVA revealed an attribution of -0.27% of the genetic variation to variability among groups and 100% variation within groups. No significant genetic differentiation was found in the within and between fishing ground variation (*F*_ST_ = -0.00279, p = 0.541).

**Table 3:** Genetic variability and Tajima’s D per fishing ground and pooled. N = number, *h* = haplotype diversity, π = nucleotide diversity.

Population	n sequences	n segregating sites	n unique haplotypes	*h* diversity	π diversity	Tajima's *D*	Tajima's *D p*-value
Tampen	27	25	20	0.961	0.006	-1.673	0.026
Bear Island	23	29	20	0.988	0.008	-1.680	0.030
Greenland	30	30	24	0.952	0.007	-1.557	0.038
Pooled	80	52	58	0.967	0.007	-1.636	0.031

## Discussion

Multidisciplinary approaches have shown to be useful to unveil differentiation of fish stocks [2,6,7,59–61]. The usefulness of parasites as biological tags can be assessed by comparing their performance against that of other markers. Hence, combinations of parasite assemblage data and parasite population genetics may potentially be successful approaches for the assessment of fish populations.

### Structure of metazoan parasite fauna

In the present study, multivariate analyses of metazoan parasite fauna successfully revealed a significant differentiation between beaked redfish from Greenland compared to redfish from Tampen, northern North Sea, and Bear Island, Barents Sea. No difference between the latter two was detected. Covariates, namely total length, Fulton’s K, and gender had no significant effect on the observed patterns. All of the identified parasites here have already been described as fish parasites that infect beaked redfish [21,62], however, larval nematode species were identified to sibling species level for the first time. The parametric ordination (LVM) showed a highest effect between Bear Island and Greenland for *C*. *nodosus*, as it occurred only in fish from Greenland, followed by *B*. *scorpii* and nematodes *A*. *simplex* s.s. and *H*. *aduncum*. The higher prevalences of *A*. *simplex* s.s. in redfish of Bear Island (P = 100%) and Tampen (P = 95%) compared to Greenland (P = 65%) are in concordance with earlier findings on these fishing grounds [35]. Cestode diversity may be underestimated as *S*. *pleuronectis* larvae could not further be determined to species level. Pielous’s evenness *E* was lower in samples from Tampen and Bear Island than from Greenland, which should reflect the dominance of the nematode species in the metazoan parasite fauna of fish in Tampen and Bear Island. Parasite species richness and taxonomic distinctness were not concordant to each other, as has been shown in previous studies [50,63,64]. The low average taxonomic distinctness (Δ^+^) and its variation (Λ^+^) in Greenland may be explained be absence of cestodes. Although the average taxonomic distinctness was highest in Bear Island, variation was high for all three fishing grounds. Some parasite genera and species, such as *C*. *nodosus*, *Hemiurus* sp., *P*. *reflexa*, and *P*. *muelleri*, were exclusively found in one or two of the locations which might be explained by differences in feeding ecology and/or habitat preferences of the intermediate and final hosts [65]. Stomach content analyses can provide insight on the main prey items in different fishing grounds [66]. For redfish, food composition and trophic interactions are difficult to assess as they often have everted stomachs when hauled to the surface from deep waters and thus, representative stomach samples *S*. *mentella* are rare [7]. Whether differences in parasite abundances can be related to a different feeding ecology could not be evaluated in the present study, as a quantitative analysis of the stomach content was not possible. Further, resulting from the eversion of stomachs during the fishing process, gastrointestinal parasites may have been released.

Parasite infra-communities were detected between fishing grounds, but special emphasis should be given on the selection of candidate parasites for use as biological tags. MacKenzie and Abaunza [67] formulated criteria for the selection of suitable parasites. These criteria encompass 1. There should be differences in prevalence or abundance among areas, 2. The presence of the parasite should be stable, 3. The parasite should not influence the condition of the host, 4. Only long-lived parasites such as encysted helminth larvae should be used for stock differentiation while short lived parasites, i.e. ectoparasites or adult helminths should be used for seasonal patterns, 5. The parasite should have a direct life cycle as otherwise more information on transmission-influencing factors is needed. Rarely can all those criteria be fulfilled and compromises need to be made. The LVM identified four parasite species as main source for clustering patterns between Greenland and the other fishing grounds Tampen and Bear Island. In the biplot, the observed pattern of *S*. *pleuronectis* sitting out from the other species can be contributed to the high intensities in few fish specimens. Although the effects were lower for the nematodes *A*. *simplex* s.s. and *H*. *aduncum* than for *C*. *nodosus*, these parasites are considered as most powerful biological tags, as they were the parasite group with highest abundances at all fishing grounds. Also the Bayes factor *K* was highest for *A*. *simplex* s.s., i.e. gave the strongest evidence of an effect to the model. Anisakids are the most commonly used parasites for stock differentiation as they are abundant and due to their large size easy to detect [18,68,69], which was confirmed in this study. The calculated probabilities to assign the correct origin of a fish according to the presence of an identified parasite species showed that a fish with *C*. *nodosus*, for example, must be from Greenland whereas one with *B*. *scorpii* cannot be. Consideration of simple presence of *A*. *simplex* s.s., this parasite species would be a poor biomarker, as it was present in 77% of all fish examined in this study. *Anisakis simplex* s.s. should therefore rather be used for comparative studies of fish assemblages that take abundances into account. Crustaceans *S*. *lumpi* and *C*. *nodosus* are often used as biological tags due to their direct life cycle; they are likely to be short-lived, but the cephalothorax of *S*. *lumpi* stays in musculature for many years [42]. *Sphyrion lumpi* was only contributing marginally to dissimilarities found among fishing grounds, probably due to the low abundances. Due to their short lifespans, Digenea and Cestoda can only be used as biological tags in a limited manner and are more suitable candidates to obtain seasonal or migration patterns [27]. Here it should be mentioned that *Scolex pleuronectis* are larval tapeworms and therefore should rather be considered in the category of having a long lifespan. Although the species *P*. *muelleri*, *Hemiurus* sp., and *P*. *reflexa* only occurred in Greenland samples while cestode *B*. *scorpii* was absent, their use as biological tags is questionable due to the aforementioned reasons. In summary, nematodes *A*. *simplex* s.s., *H*. *aduncum*, *P*. *decipiens s*.*s*., and crustaceans *S*. *lumpi* and *C*. *nodosus* are useful for stock differentiation, while *B*. *scorpii*, *P*. *muelleri*, *Hemiurus* sp., and *P*. *reflexa* are candidates for migration and seasonal patterns.

### Haplotype structuring

When choosing biological tags to separate fish stocks, genetic structure of parasite populations potentially identify host stock structure or host migration patterns more accurately than classic parasite abundance measurements [69]. Commonly used markers for population structure analyses are mitochondrial genes such as *cox2* (cytochrome oxidase c) with their high substitution rate and maternal inheritance [2]. Here, no geographic separation could be identified in *A*. *simplex* s.s. using *cox2* cytochrome marker to investigate the haplotype structure from the three fishing grounds. These findings are supported by other studies on *Anisakis* mtDNA structure from Adriatic, Atlantic, and Pacific fish [24,29,70]. Haplotype diversity was high with 86% of unique haplotypes and *A*. *simplex* larvae from all three fishing grounds shared the same (most abundant) haplotype. The generally low nucleotide diversity (0.007) was slightly lower in comparison with nematode larvae isolated from sardines from the California current (0.018) [29], the same as in Adriatic fish populations (0.007) [70], and higher than in cetacean hosts in the Adriatic population (0.0045) [71]. High haplotype and low nucleotide diversity stems mainly from single nucleotide mutations. Furthermore, mean pairwise divergence and *F*_ST_ were low, and consequently no genetic distinction between *A*. *simplex* larvae could be detected which suggests a lack of genetic structuring and the existence of a single population of *A*. *simplex* in the North East Atlantic. AMOVA revealed a high intra-population variation which may be explained by high substitution rates in mtDNA, high gene flow and large effective population sizes [72]. This could be the case for *A*. *simplex* s.s. [24]. Both Tajima’s D and Fu’s F were highly negative, indicating recent demographic processes with recent range expansion as shown by the excess of rare nucleotide site variants and haplotypes than would be expected under neutrality [54,55,70]. The non-existence of haplotype structuring over large distances may be explained by high gene flow of *Anisakis* populations among fishing grounds. Parasite genetic population structuring is usually considered to be influenced by a fragmented nature of the habitat, and limited dispersal over long distances [73]. The main determinant for haplotype panmixia is likely to be derived from high gene flow between geographically distant *Anisakis* larvae through the dispersal by both paratenic fish and cetacean final hosts, which help to overcome oceanic barriers that exist in the North East Atlantic current system [24,44,74].

The usefulness in the application of *A*. *simplex* haplotypes in order to distinguish fish populations is limited. Biogeographic structuring of *Anisakis* species based on mtDNA markers may only be found over large oceanic distances. Parasites that have a high dispersal and thus high gene flow exhibit also high genetic diversity within populations [75]. Hence, the choice of parasite species with less motile hosts and consequently higher restrictions in gene flow for biological tags may be recommended. Other genetic markers such as non-coding fast evolving nuclear markers (SNPs, microsatellites) that are hypervariable may be more suitable for detection of genetic differentiation among individual parasites within shorter time-scales as well as differences within regions.

## Conclusions

In the present study, we compared two methods, metazoan parasite assemblage and *A*. *simplex* haplotype structure, to examine whether stocks of *S*. *mentella* can be separated in the North East Atlantic region. Overall, no sub-structuring of *S*. *mentella* in the northern North Sea and Barents Sea was detected. Four parasite species were identified as best candidates to distinguish redfish from three fishing grounds. Anisakid nematode larvae, especially *A*. *simplex* s.s. were considered as major candidates to discriminate stocks as they contributed to the total difference of parasite assemblages among fishing grounds and were most abundant. This study underlines, on the one hand, the importance of multivariate analyses as a tool for the evaluation of parasite infra-communities and to determine the most relevant candidate species for biological tags. On the other hand, it shows that the sole examination of *A*. *simplex* s.s. on a population genetic level is not sufficient to discriminate fish stocks. As the revealed haplotype panmixia was in line with results from previous studies from different locations and fish species, haplotype analysis of *A*. *simplex* larvae based on *cox2* cytochrome markers is not considered as useful tool for population/ host stock differentiation.
